# Qu-Yu-Jie-Du Decoction Ameliorates Dextran Sulfate Sodium-Induced Colitis in Mice by Modulation of Neutrophils and Macrophage Infiltration

**DOI:** 10.1155/2022/3762591

**Published:** 2022-12-06

**Authors:** Hongwei Zhao, Lingling Sun, Xi Xiao, Jietao Lin, Cui Shao, Lizhu Lin

**Affiliations:** Department of Oncology, The First Affiliated Hospital, Guangzhou University of Chinese Medicine, Guangzhou 510405, China

## Abstract

**Background:**

Inflammatory bowel disease (IBD) is becoming a global disease. A percentage of IBD patients will not react to therapy or will lose their response. Qu-Yu-Jie-Du Decoction (QYJD) is a traditional Chinese medicine formula commonly used for intestinal diseases. It has been reported that QYJD has an anti-inflammatory effect, but the mechanism is not fully understood. In this study, we mainly evaluated the anti-inflammatory effect of QYJD and explored the possible mechanisms.

**Methods:**

Twenty-four BALB/*c* mice were randomly divided into 4 groups according to their body weight, namely, the control group, the dextran sulfate sodium (DSS) group, the DSS + QYJD group, and the QYJD group. Mice were given 3% DSS drinking water freely, and at the same time, mice were given normal saline or QYJD (4.44 mg/g/d), respectively. Mental state, faeces, and weight were recorded every day. On the 10th day, the mice were sacrificed and collected for investigation. The length of the mice colon was measured. Histological analysis was used to detect the morphological changes in the colon. Immunohistochemistry was used to measure the infiltration of macrophages (F4/80, CD163) and neutrophils (Ly6G). Colorimetry was used to detect the myeloperoxidase (MPO) activity of colon tissues. ELISA was utilized to detect associated inflammatory cytokines and chemokines in colon tissues.

**Results:**

QYJD alleviated the weight loss and colitis symptoms of mice caused by DSS. QYJD fought against the shortening of the intestine caused by DSS; that is, it improved the decline of intestinal compliance in mice and had a protective effect on colon tissues. The mechanisms were related to downregulating macrophages and neutrophils in colon tissues of infiltration. Besides, QYJD simultaneously reduced the activity of myeloperoxidase activity (MPO) and the contents of IL-1*β*, IL-6, TNF-*α*, TGF-*β*, CCL2, and CXCL2 in colon tissues.

**Conclusions:**

QYJD can ameliorate DSS-induced colitis in mice and the mechanism is connected with a reduction in neutrophil and macrophage infiltration.

## 1. Introduction

Inflammatory bowel disease (IBD) is becoming a global disease in the twenty-first century. According to statistics, there are more than 2 million IBD patients in North America, 3.2 million in Europe, and millions more around the world. The incidence rates of IBD in newly industrialized countries have been on the rise in recent years with the westernization of lifestyle [1]. Although the etiology of IBD is not completely clear, it may result from predisposing genetic mutations, complex environmental factors, gut microbiota, and immune responses [2–4]. Crohn's disease (CD) and ulcerative colitis (UC) are known as the two major forms of this disease. CD leads to transmural inflammation and occurs in any part of the gastrointestinal tract continuously. CD commonly results in complications such as abscesses, fistulas, and strictures. UC is appertaining to mucosal inflammation and is limited to the colon in an uninterrupted pattern [4]. IBD presents with chronic inflammation, a relapsing and remitting clinical course, the requirement for lifelong medication or even surgery, and decreased quality of life. It is well recognized that patients with IBD are at an increased risk of developing colorectal cancer (CRC) and extra-intestinal malignancies, primarily secondary to chronic inflammation state and immunosuppressive therapies [5, 6]. The main treatments for IBD are aminosalicylates, short courses of steroids for severe flares, and escalation to immunomodulators. Despite the fact that there are numerous therapeutic alternatives for the treatment of IBD, a portion of patients fail to respond or lose their response to medication [7]. Meanwhile, healthcare costs are dramatically increased. Thus, new therapeutic strategies for IBD are urgently needed.

Neutrophils and macrophages are essential innate immune cells playing a variety of roles in the host's pathogen defense [8]. They are able to generate effector molecules such as granular proteins, oxidants, and cytokines, among others [9, 10]. When inflammation occurs, neutrophils are the first to be recruited and are outfitted with powerful microbicidal activity. Neutrophils can release chemoattractants such as cathepsin G and azurocidin, which aid in the recruitment of other immune cells [11]. Then, macrophages are recruited, which have the ability to digest antigens and present them to other immune cells, enabling them to interact with the adaptive immune system [12]. Neutrophil chemoattractants such as CXCL1, CXCL2, and monocyte chemoattractant protein-1(MCP-1) are produced when tissue-resident macrophages are activated [13, 14]. Evidently, neutrophils and macrophages organize a successful immune response against pathogens [15].

Qu-Yu-Jie-Du Decoction (QYJD) is an empirical formula for intestinal diseases based on the classic works of physicians of all dynasties and years of clinical experience, which has the effect of removing blood stasis and detoxifying. The formula is composed of eight kinds of Chinese medicinal materials, including Taoren (*Semen Persicae*, peach seed), Kushen (*Radix Sophorae Flavescentis*, light yellow sophora root), tufuling (*Rhizoma Smilacis Glabrae*, glabrous greenbrier rhizome), Diyu (*Radix Sanguisorbae*, garden burnet root), Yiyiren (*Semen Coicis*, coix seed), Huaihua (*Flos Sophorae*, pagoda tree flower), Pugongying (*Herba Taraxaci*, dandelion), and Tubiechong (*Eupolyphaga Seu Steleophaga*, ground beetle). QYJD is effective in alleviating colitis in C57BL/6J mice by upregulating Nrf2 and HO-1 mRNA levels and antioxidant stress responses. However, whether QYJD regulates neutrophils or macrophage inflammatory response in IBD is largely unknown [16].

Dextran sodium sulfate (DSS), a water-soluble sulfated polysaccharide, is the most widely used to induce a mouse model of colitis. The molecular weight of DSS used for induction of colitis is 36–50 kD. DSS is toxic to the colon epithelium and can induce erosion that compromises barrier integrity, resulting in increased permeability of the colon epithelium and diffusion of proinflammatory intestinal substances, such as bacteria and their items, into the underlying tissues. In addition, DSS has an anticoagulant character that exacerbates intestinal bleeding. The DSS colitis model is widely used in IBD research because of its quickness, effortlessness, and numerous similarities with human UC [7, 17, 18]. In this current study, we validated that QYJD can ameliorate acute colitis in dextran sulfate sodium (DSS)-induced mouse models. We also demonstrated that the alleviation of colitis by QYJD was related to the modulation of neutrophils and macrophage infiltration.

## 2. Materials and Method

### 2.1. Animal Care

Female BALB/c mice, 6–8 week-old, weighing 17–20 g, were purchased from Beijing Weitong Lihua Experimental Animal Technology Co., Ltd. (Production License: SCXK (Beijing) 2016–0006). They were fed with standard pathogen-free food and water, housed under a 12-hour light/dark cycle in a humidified room at 25°C. The mice were kept in the Experimental Animal Center, Guangdong Pharmaceutical University. All animal studies were approved by the Ethics Committee of Animal Experiments of Guangdong Pharmaceutical University (No. gdpulacSPF2017265).

### 2.2. Herbal Preparation

All herbs were purchased from the First Affiliated Hospital of Guangzhou University of Chinese Medicine (China). The components of QYJD are shown in Table 1. According to the prescription, we weighed the crude drugs and added water to exceed 5 cm of the drug surface to soak for 30 minutes. We boiled the mixture with gentle heat for 40 minutes and then collected the decoction. We added water to the drug residue to 1 cm above the surface and boiled it gently for 30 minutes again. We obtained the QYJD extract by collecting and mixing the two decoctions. The extract was concentrated to 1.8 g/mL by a rotary evaporator. The concentrates were made into a freeze-dried powder with a vacuum freeze-drying machine in a cold trap at −50°C. The freeze-dried powder was sealed at −20°C. The final weight ratio of crude drug to lyophilized powder was 9.756. According to the LC/MS chromatogram of QYJD, matrine and oxymatrine are the two main components of QYJD [16].

### 2.3. Reagents and Apparatus

DSS (MW 36000–50000) was purchased from MP Biomedicals Co., Ltd. (OH, USA); the myeloperoxidase assay kit was obtained from Nanjing Jiancheng Bioengineering Institute (Nanjing, China); the CCR2 antibody was from Abcam (Cambridge, MA); F4/80, Ly6G, CD163, and all secondary antibodies were obtained from Wuhan Service Biotechnology Co., Ltd. (Wuhan, China); IL-1*β*, IL-6, IL-12, TNF-*α*, TGF-*β*1, CXCL1, MCP-1, and CXCL2 mini samples and ELISA kits were purchased from Cloud-Clone Corp. (Wuhan, China). The freezer dryer was from Ningbo Xinzhi Biotechnology Co., Ltd. (Ningbo, China); enzyme standard instrument was purchased from Thermo Fisher (Shanghai) Instruments Co., Ltd. (Shanghai, China); automated tissue homogenization was from MP Biomedicals Co., Ltd. (OH, USA). A single-beam UV-Vis spectrophotometer was purchased from Shanghai Xinmao Instrument Co., Ltd. (Shanghai, China).

### 2.4. Induction of Colitis and Treatment

24 BALB/C mice were randomly divided into 4 groups according to their body weight: (1) control group (without DSS and QYJD); (2) DSS group; (3) DSS + QYJD group; and (4) QYJD group (without DSS). Colitis was induced by the administration of 3% (w/v) DSS in drinking water for 9 days. At the same time, mice of the control group and DSS group were given intragastric administration of normal saline once daily. Mice of the DSS + QYJD group and QYJD group were simultaneously given intragastric administration of 4.44 mg/g QYJD once daily. The volume of gavage was 10 *μ*L/g. The mental state of the mice was observed every day, including whether there was back arching, slow activity, or reduced eating. The weight of the mice was measured, and the stool situation and hematochezia of the mice were recorded daily. If the mice did not defecate immediately, continue to observe for at least 15 minutes. On the 10th day, the mice were sacrificed, and colon tissue samples were gathered.

### 2.5. Disease Activity Index (DAI)

The DAI was comprehensively evaluated by weight change, stool texture, and hematochezia of mice, reflecting the severity of colitis [17, 19]. DAI of mice was calculated according to Table 2. The disease activity index = (score of weight loss + score of consistency + score of bleeding)/3.

### 2.6. Histological Analysis

The colon tissue (about 3 cm) near the anal end of mice was taken and fixed in 4% paraformaldehyde for 24 h with the method of swiss-rolls. After dehydration, the tissues were embedded in paraffin cut into 4 *μ*m layers and stained with hematoxylin and eosin (H&E) for histological evaluation [20]. Histological scoring was calculated by adding the six evaluations as follows [21, 22]:

1. Ulcer or erosion (0 = none; 1 = less than 3 mm; 2 = greater than 3 mm);
2. Edema (0 = none; 1 = yes);
3. Hyperplasia (0 = normal; 1 = mild; 2 = moderate; 3 = heavy);
4. Degree of inflammation (0 = none; 1 = slight; 2 = mild; 3 = moderate; 4 = heavy);
5. Degree of crypt damage (0 = complete crypt; 1 = cystic dilation; 2 = loss of one-third of the base; 3 = two-thirds of the crypt of the base is lost; 4 = the entire crypt was lost, but the surface epithelium remained intact; 5 = loss of crypt and surface epithelium);
6. Lesion depth (0 = none; 1 = mucosal layer; 2 = submucosa; 3 = muscular layer; 4 = serous layer).

### 2.7. Immunohistochemistry

The slides were dewaxed using xylene, different concentrations of ethanol, and rinsed with water. Then, the slides were placed into citric acid antigen repair buffer and heated in a pressure cooker for tissue antigen repair. We put the slices in 3% H_2_O_2_ solution away from light for 25 minutes at room temperature to block endogenous peroxidase. 3% BSA was dropped onto the tissue to seal. Remove the blocking solution and drop the primary antibody onto the glass slide (dilution ratios of antibodies: F4/80 antibody 1 : 500; CD163 antibody 1 : 250; ly6G antibody 1 : 400; CCR2 antibody 1 : 250). The slides were placed in a wet box and incubated overnight at 4°C. And then, the slides were incubated with HRP-labeled goat anti-rabbit IgG for 50 minutes at room temperature. DAB color solution was added to develop color. The nucleus was restained with hematoxylin for 3 minutes. The slides were dehydrated and sealed. We used Image-Pro Plus 6.0 software to select the brown-yellow color with positive expression as the standard to judge the positivity of all photos, analyze the cumulative optical density (IOD) of the images and the pixel Area of the tissues, and calculate the areal density. Areal density = IOD/Area.

### 2.8. Measurement of Myeloperoxidase (MPO) in Colon

The activity of myeloperoxidase (MPO) in colon tissue was determined by colorimetry to evaluate the number and function of neutrophils in colon tissues. A homogenate of 5% was prepared by adding homogenate medium at a ratio of 1 : 19 (w/v). We added reagents according to the manufacturer's instructions and then used a spectrophotometer to detect the absorbance of each tube at 460 nm. The MPO activity is evaluated by the following formula: The MPO activity (U/tissue wet weight (g))  = (determination OD value−control OD value)/(11.3 × sample weight (g)).

### 2.9. Determination of Cytokines and Chemokines Levels in Colon

The cytokines and chemokines levels in the colon were measured by ELISA kits according to the manufacturer's instructions. The tissue homogenates were prepared by adding lysate at a ratio of 1 : 100 (100 mg tissue and 1 mL tissue lysate). Homogenates were centrifuged (12000 rpm, 4°C, 20 minutes) to collect the supernatants. Supernatants were employed to estimate cytokines and chemokines. Draw the standard curve according to the concentration of the standard substance and the optical density value (OD). The OD values of each well at 450 nm were measured with a microplate reader with Sigma Plot 12.0 software. The concentration values of the samples to be tested were calculated according to the standard curve, and the results were represented as pg/mg of tissue.

### 2.10. Statistical Analysis

IBM SPSS Statistics 25.0 (Standford, CA, USA) and Graphpad Prism 6.0 software were used for experimental data analysis and mapping. Continuous variable data were represented by the mean ± standard deviation. Continuous measurement data was analyzed by repeated ANOVA. For the comparison of multiple groups, one-way ANOVA was used for normal distribution data. LSD and Dunnett T3 were used for pairwise comparison between groups. Kruskal–Wallis H test was used for statistical analysis of skewness distribution data. Data from the two groups were analyzed utilizing the independent sample *T* test or Mann–Whitney U rank-sum test. *P*  <  0.05 was considered to be statistically significant.

## 3. Results

### 3.1. QYJD Attenuated DSS-Induced Colitis

**Figure 1:**
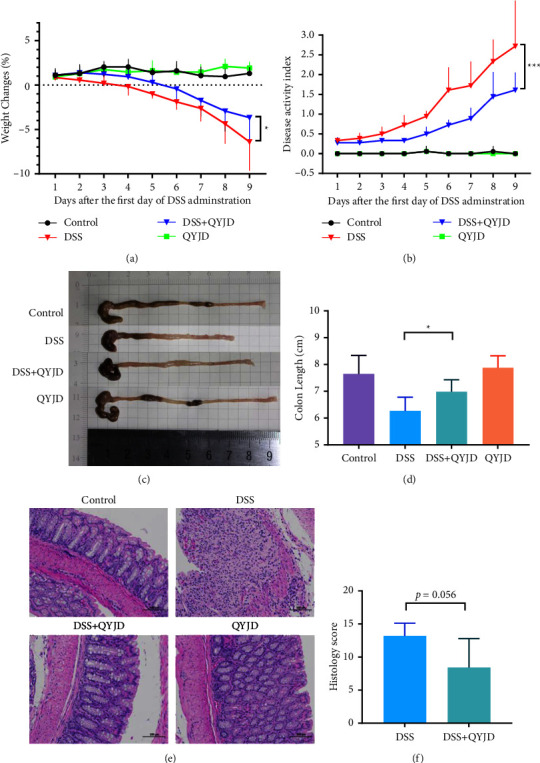
QYJD ameliorated DSS-induced murine colitis. (a) Body weight changes in each group during treatment. (b) Effect of QYJD on DSS-induced changes in disease activity index (DAI). (c) Representative images of the colon of each group. (d) Effect of QYJD on DSS-induced changes in colon length. (e) Representative photomicrograph of colonic tissue stained with hematoxylin and eosin of each group. (200×, 100 *μ*m). Control: clear structure of each layer, complete epithelium of mucosal layer, abundant and compact intestinal gland, normal cell morphology, and no obvious inflammatory reaction in colon tissue. DSS: large area ulcers, loss of the mucosal epithelial cells, loss of intestinal gland structure, loss of goblet cells, hyperplasia of connective tissue, injury and invasion of the submucosa, a large number of inflammatory cells were found in the mucosal layer and submucosa. DSS + QYJD: the mucosal layer was damaged, the local intestinal gland structure disappeared, connective tissue hyperplasia, and a small amount of inflammatory cell infiltration. QYJD: displayed similar morphology to the control group. (f) Comparison of histopathological score between the DSS group and DSS + QYJD group. Data are shown as mean ± SEM. ^*∗*^*P*  < 0.05, ^*∗∗*^*P* <  0.01, ^*∗∗∗*^*P* < 0.001.

In order to study the therapeutic effect of QYJD on DSS-induced colitis in BALB/c mice, we evaluated from weight change, disease activity index (DAI), colon length, and histological analysis. As shown in Figure 1(a), the body weight of the DSS group decreased significantly compared with the control group, *P* <  0.001. The weight loss degree of the DSS + QYJD group was less than that of the DSS group, *P*=0.015. These results demonstrate that DSS could cause weight loss in mice, QYJD could reduce the degree of weight loss in mice caused by DSS, while QYJD alone had no significant effect on the weight of mice. The severity of colitis was evaluated daily using the disease activity index (DAI) scoring system, which combined weight loss, stool consistency, and bleeding scores. The DAI of the DSS group was significantly higher than that of the control group, *P* <  0.001; The DAI of the DSS + QYJD group was significantly lower than that of the DSS group, *P* <  0.001. There was no statistical difference between the control group and the QYJD group, *P*=0.941 (Figure 1(b)). Colon length, an indicator of intestinal permeability, was significantly shortened in the DSS group than that in the control group, *P* <  0.001. While the colon length of mice in the DSS + QYJD group was significantly greater than the DSS group, *P*=0.041 (Figures 1(c) and 1(d)). QYJD could alleviate DSS-induced colon length shortening.

To determine the status of the colon, a histological analysis was performed after colon tissue was stained with hematoxylin and eosin. As shown in Figure 1(e), in the DSS group, large area ulcers were found in colon tissue, loss of mucosal epithelial cells, loss of intestinal gland structure, loss of goblet cells, hyperplasia of connective tissue, and a small amount of blood vessel congestion, injury, and invasion of the submucosa, and a large number of inflammatory cells were found in the mucosal layer and submucosa. In the DSS + QYJD group, the mucosal layer of colon tissue was damaged, the local intestinal gland structure disappeared, connective tissue hyperplasia was accompanied by a small amount of inflammatory cell infiltration, and the damage did not invade the submucosa. Mice in the control group and QYJD group had a clear structure of each layer of colon tissue, complete epithelium of mucosal layer, abundant and compact intestinal gland, normal cell morphology, and no obvious inflammatory reaction in colon tissue. Furthermore, the histological score of the DSS + QYJD group was lower than the DSS group (*P*=0.056), but the differences were not significant (Figure 1(f)).

### 3.2. QYJD Decreased Neutrophil Infiltration and the Activity of Myeloperoxidase Activity (MPO)

**Figure 2:**
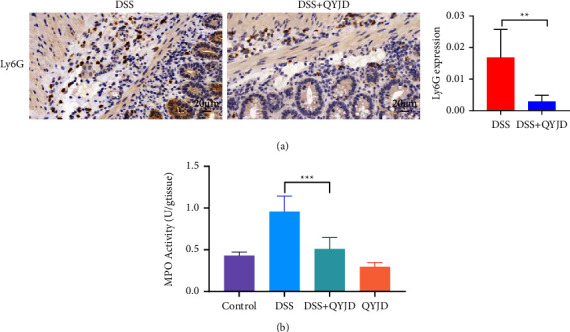
QYJD decreased neutrophil infiltration and the activity of MPO in colon tissues of mice with colitis. (a) Expression of Ly6G in colon tissue detected by immunohistochemistry (400×). (b) MPO activity in colon tissue. Data are expressed as mean ± SEM, ^*∗*^*P*  <  0.05, ^*∗∗*^*P*  <  0.01, ^*∗∗∗*^*P*  <  0.001.

In order to evaluate the effect of QYJD on neutrophils in the colon tissue of DSS-induced colitis mice, we used immunohistochemistry to detect the expression of Ly6G in colon tissue. As shown in Figure 2(a), the expression of Ly6G in the DSS + QYJD group was significantly lower than that in the DSS group (*P*=0.001). The activity of myeloperoxidase (MPO) represents the infiltration of neutrophils and stress intensity to injury. Compared with the control group, the MPO activity of colon tissue in the DSS group was significantly increased (*P* <  0.001). The MPO activity in the DSS + QYJD group was significantly lower than that in the DSS group (*P* <  0.001) (Figure 2(b)).

### 3.3. QYJD Reduced Macrophage Infiltration

**Figure 3:**
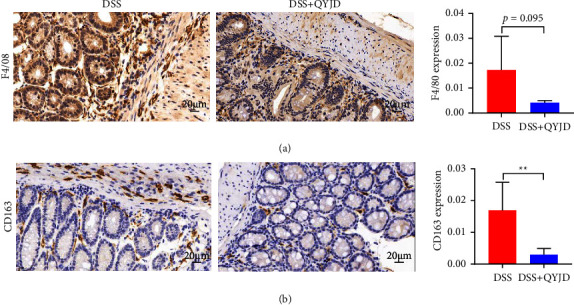
QYJD inhibited macrophage infiltration in colon tissues of mice with colitis. (a) Expression of F4/80 in colon tissue detected by immunohistochemistry (400×). (b) Expression of CD163 in colon tissue detected by immunohistochemistry (400×). Data are expressed as mean ± SEM, ^*∗*^*P* < 0.05, ^*∗∗*^*P* < 0.01, and ^*∗∗∗*^*P* < 0.001.

F4/80 and CD163 were used as markers of macrophage. The expression level of F4/80 in the DSS + QYJD group was lower than that in the DSS group in trend, but there was no significant statistical difference (*P*=0.095) (Figure 3(a)). The expression level of CD163 in the DSS + QYJD group was significantly lower than that in the DSS group (*P*=0.001) (Figure 3(b)).

### 3.4. QYJD Regulated the Expression of Inflammation Cytokine

**Figure 4:**
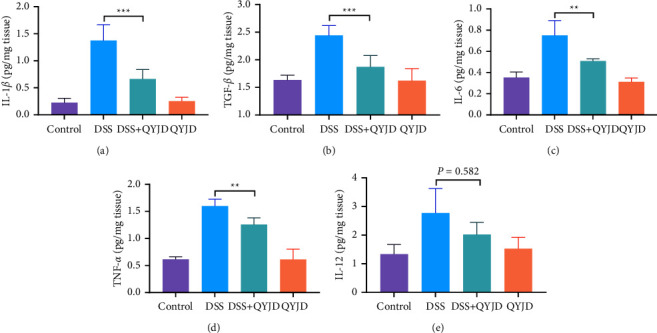
Effect of QYJD on macrophage and neutrophil-related inflammation cytokines. (a–d) The protein levels of macrophage-associated cytokine IL-1*β*, IL-6, TNF-*α*, and TGF-*β* in colonic tissue. (e) The protein level of neutrophil-associated cytokine IL-12 in colonic tissue. The protein levels of cytokines were quantified by ELISA kits. ^*∗*^*P*  <  0.05, ^*∗∗*^*P*  <  0.01, and ^*∗∗∗*^*P*  <  0.001.

To further investigate the mechanism of QYJD on DSS-induced colitis in mice, we measured macrophage-related and neutrophil-related inflammation cytokines in the colon tissues of mice. Compared with the control group, DSS could significantly increase the protein levels of inflammation cytokines IL-1*β*, IL-6, TNF-*α*, TGF-*β,* and IL-12 in colonic tissues. As shown in Figure 4, the protein levels of Il-1*β*, TGF-*β*, TNF-*α,* and IL-6 in the DSS + QYJD group were significantly lower than those in the DSS group.

### 3.5. QYJD Regulated the Chemokines Expression

**Figure 5:**
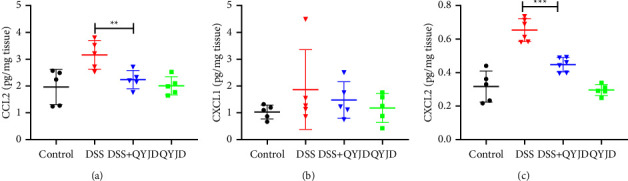
Effect of QYJD on macrophage and neutrophil-related chemokines. (a) The protein levels of CCL2 in colonic tissue. (b) The protein level of CXCL1 in colonic tissue. (c) The protein level of CXCL2 in colonic tissue. The protein levels of cytokines were quantified by ELISA kits. ^*∗*^*P*  <  0.05, ^*∗∗*^*P*  <  0.01, and ^*∗∗∗*^*P*  <  0.001.

Compared with the control group, DSS could significantly increase the protein levels of CCL2 and CXCL2. The content of chemokines CCL2 and CXCL2 in the DSS + QYJD group were significantly lower than those in the DSS group. The results showed that QYJD could reduce the content of chemokines CCL2 and CXCL2 in colonic tissue of mouse models of colitis induced by DSS. While there was no significant difference in CXCL1 expression among all groups (Figure 5).

## 4. Discussion

Our study indicated that QYJD could ameliorate DSS-induced colitis in mice by reducing the infiltration of neutrophils and macrophages. As for our result, we assessed the anti-inflammatory effect of QYJD by observing the degree of weight loss, the DAI score, colon length, and histopathology. The activity of myeloperoxidase (MPO) represents the infiltration of neutrophils and stress intensity to injury [23, 24]. Impressively, DSS can significantly increase MPO activity in the colon tissue of mice, and QYJD can effectively reduce MPO activity in the colon tissue of mice.

To study the anti-inflammatory mechanism of QYJD, we first observed the effect of QYJD on congenital immune cells such as macrophages and neutrophils in colon tissue. Macrophages play a central role in maintaining intestinal homeostasis by engulfing necrotic cell debris and pathogens [25]. Neutrophils are important effectors of antimicrobial immunity, secreting matrix metalloproteinases and myeloperoxidase during inflammation. The infiltration of macrophages and neutrophils in the colon tissues of each group was detected by immunohistochemistry. F4/80 is a surface marker protein of macrophages, representing the infiltration of macrophages. CD163 was highly expressed in M2 macrophages. Ly6G is mainly expressed on the membrane surface of neutrophils. We found that compared with the DSS group, QYJD could significantly reduce the expression levels of CD163 and Ly6G in colon tissues. The expression of F4/80 in the treatment group of QYJD showed a downward trend, but there was no significant difference between the two groups, which might be caused by the small sample size.

When the colonic mucosa is damaged by DSS and other pathogenic factors, many inflammatory cytokines are increased. Among these cytokines, IL-1*β*, IL-6, and TNF-*α* play essential roles in intestinal inflammation [26]. Furthermore, we also measured TGF-*β*, which plays important roles in growth and development, inflammation and repair, and immunity [27]. In this study, we used ELISA to detect the contents of cytokines IL-1*β*, IL-6, TNF-*α*, IL-12, and TGF-*β* in mouse intestinal tissue, and we found that QYJD could significantly reduce the contents of IL-1*β*, IL-6, TNF-*α,* and TGF-*β* in colon tissue of mice with colitis.

To further consolidate the effect of QYJD in regulating neutrophils and macrophages to block inflammation cascades, we tested related chemokines. Chemokines belong to the *G* protein-coupled receptor superfamily of cytokines, which are related to the migration and activation of leukocytes and play a major role in maintaining inflammation [28]. CCL2 is also called monocyte chemoattractant protein-1(MCP-1). CCL2/MCP-1 is produced by dendritic cells, macrophages, fibroblasts, keratinocytes, and other cells. The function of CCL2/MCP-1 is to lead monocytes to leave the blood and differentiate into macrophages in tissues, promote the release of histamine from basophils, and improve Th2 activity [29, 30]. In addition, CCL2 has chemotactic effects on T cells, mast cells, basophils, and natural killer cells [31, 32]. Elevated CCL2 can be detected in the intestinal mucosal tissue of patients with IBD [33]. CXCL2 is a protein encoded by proto-oncogenes derived from monocytes, fibroblasts, and endothelial cells. CXCL2 has the function of chemotaxis to neutrophils, initial T lymphocytes, and fibroblasts. In our study, we found that QYJD down-regulated the expression of CCL2 and CXCL2 in colon tissues, resulting in less locally infiltrated neutrophils and macrophages.

Strengths of our study include that the mice were randomly assigned by weight, and the basic condition of mice in each group was the same. Besides, we observed the effects of QYJD on a variety of cytokines and chemokines in the colon tissues of mice. This reflects the characteristics of multipathway and multitarget of the TCM, providing ideas for follow-up studies and having certain reference values, although there are still some shortcomings, such as insufficient mechanism digging. But we have tested it multiple times and proved that QYJD could reduce DSS-induced intestinal inflammatory response in mice. Inflammation affects cancer invasion, epithelial-mesenchymal transformation, and cell migration at many levels [34]. Inflammation, inflammatory cytokine release, and inflammatory cell infiltration are regarded as major contributors to the initiation and development of UC and its associated cancer [35]. This study offered an approach to studying the effect of QYJD on the malignant tumor. In future studies, we will verify by enlarging the sample size and exploring from the perspective of neutrophils and macrophages, considering its significant changes and the important role of the inflammatory process.

## 5. Conclusions

QYJD has the effect of inhibiting colitis in mice, and its mechanism is related to the regulation of the inflammatory immune system. QYJD can mitigate the weight loss and enteritis symptoms of mice caused by DSS. It fights against the shortening of the intestine brought about by DSS, improves the decline of intestinal compliance in mice, and has a protective effect on colon tissue. The mechanism is related to the regulation of IL-1*β*, TGF-*β*, IL-6, TNF-*α,* and other cytokines in colon tissue by QYJD, and the reduction of chemokine CCL2 and CXCL2, as well as the reduction of neutrophils and macrophages infiltration in colon tissue.

## Figures and Tables

**Table 1:** The components of QYJD.

Chinese name	Latin name	Plant part	Occupied percent (%)	Weight of crude drugs in QYJD (g)
Taoren	*Semen Persicae*	Peach seed	8.3	12
Kushen	*Radix Sophorae Flavescentis*	Light yellow sophora root	8.3	12
Tufuling	*Rhizoma Smilacis Glabrae*	Glabrous greenbrier rhizome	16.7	24
Diyu	*Radix Sanguisorbae*	Garden burnet root	10.4	15
Yiyiren	*Semen Coicis*	Coix seed	20.8	30
Huaihua	*Flos Sophorae*	Pagoda tree flower	10.4	15
Pugongying	*Herba Taraxaci*	Dandelion	20.8	30
Tubiechong	*Eupolyphaga Seu Steleophaga*	Ground beetle	4.2	6

**Table 2:** Disease activity index.

Score	Weight loss (%)	Stool consistency	Bleeding
0	≤1	Normal	Negative
1	>1, ≤5	Soft	—
2	>5, ≤10	Loose	—
3	>10, ≤15	Pasty	Blood traces in the stool are clearly visible
4	>15	Diarrhea	Gross bleeding

## Data Availability

Data are available upon request by sending an e-mail to the author Hongwei Zhao (zhaohongwei129@163.com).

## Supplementary Materials

Supplementary Materials The authors did a pilot experiment earlier, which was a dose-dependent design. Details are as follows: 25 BALB/c mice were randomly divided into 5 groups according to their body weight: (1) control group (0.9% NaCl, without DSS and QYJD); (2) DSS group (0.9% NaCl); (3) DSS + QYJD × 1/2 group (the concentration of QYJD was 1.11 mg/g/d); (4) DSS + QYJD × 1 group (the concentration of QYJD was 2.22 mg/g/d); (5) DSS + QYJD × 2 group (the concentration of QYJD was 4.44 mg/g/d). Colitis was induced by the administration of 3% (w/v) DSS in drinking water for 7 days. At the same time, mice were given intragastric administration of normal saline or different doses of QYJD once a day. The volume of gavage was 10 *μ*L/g. The mental state of the mice was observed daily, including whether there was slow activity, reduced eating, and back arching. The weight was measured; the stool situation and hematochezia of the mice were recorded every day. According to the scores of the disease activity index (DAI) and weight loss (%) of the mice, we found that twice the equivalent dose of QYJD had a better effect on attenuating DSS-induced colitis. Therefore, twice the equivalent dose (4.44 mg/g/d) of QYJD was used in the subsequent formal experiment (Figure S1).
