# Supplementary material for: Qu-Yu-Jie-Du Decoction Ameliorates Dextran Sulfate Sodium-Induced Colitis in Mice by Modulation of Neutrophils and Macrophage Infiltration
Source: Evid Based Complement Alternat Med. 2022 Dec 6;2022:3762591. doi: 10.1155/2022/3762591 (PMC9747316; doi:10.1155/2022/3762591)
Supplement: Supplementary Materials — The authors did a pilot experiment earlier, which was a dose-dependent design. Details are as follows: 25 BALB/c mice were randomly divided into 5 groups according to their body weight: (1) control group (0.9% NaCl, without DSS and QYJD); (2) DSS group (0.9% NaCl); (3) DSS + QYJD × 1/2 group (the concentration of QYJD was 1.11 mg/g/d); (4) DSS + QYJD × 1 group (the concentration of QYJD was 2.22 mg/g/d); (5) DSS + QYJD × 2 group (the concentration of QYJD was 4.44 mg/g/d). Colitis was induced by the administration of 3% (w/v) DSS in drinking water for 7 days. At the same time, mice were given intragastric administration of normal saline or different doses of QYJD once a day. The volume of gavage was 10 μL/g. The mental state of the mice was observed daily, including whether there was slow activity, reduced eating, and back arching. The weight was measured; the stool situation and hematochezia of the mice were recorded every day. According to the scores of the disease activity index (DAI) and weight loss (%) of the mice, we found that twice the equivalent dose of QYJD had a better effect on attenuating DSS-induced colitis. Therefore, twice the equivalent dose (4.44 mg/g/d) of QYJD was used in the subsequent formal experiment (Figure S1). [file 3762591.f1.docx]

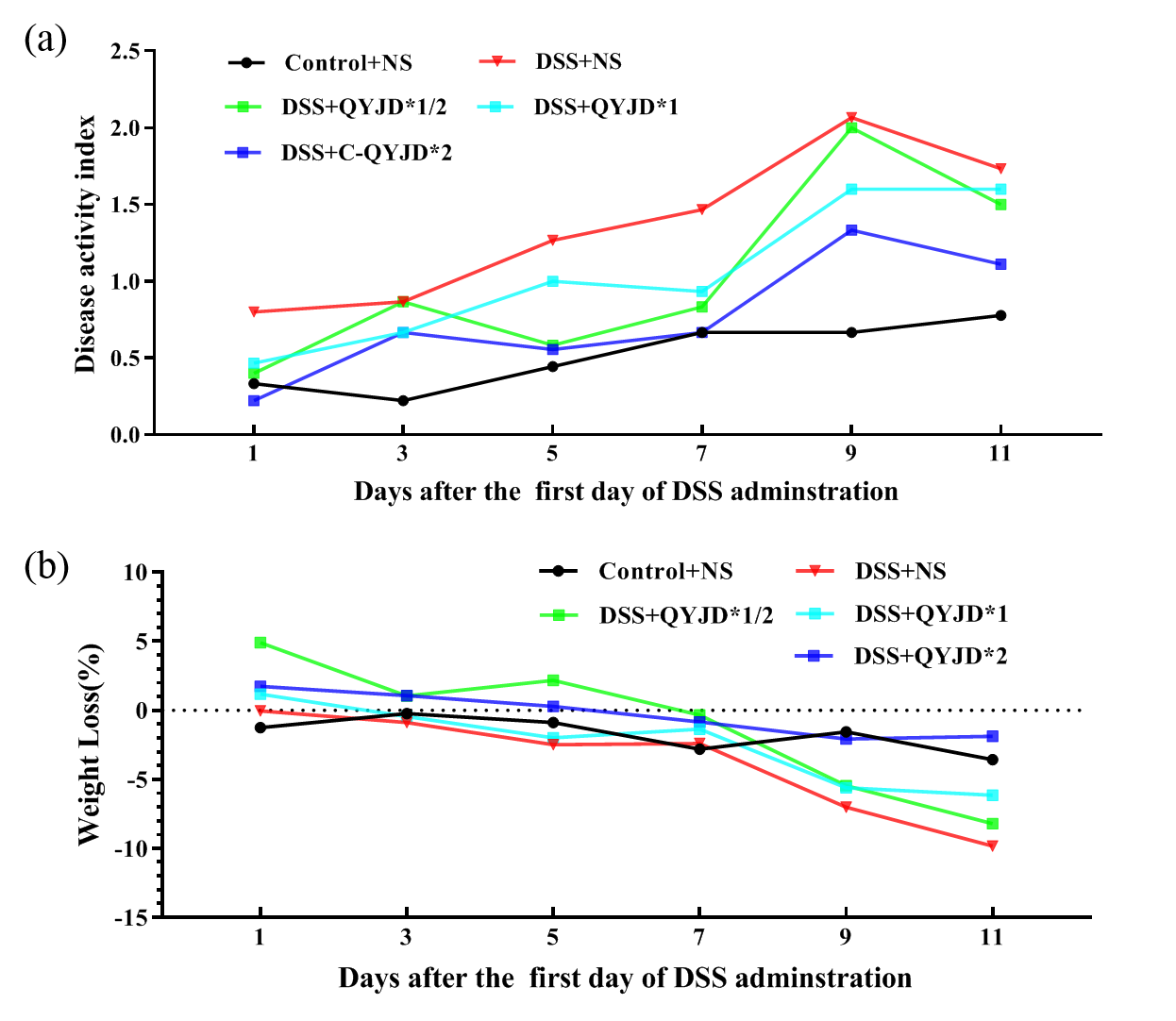


Figure S1: QYJD ameliorated DSS-induced murine colitis. (a) Effect of QYJD on DSS-induced changes in disease activity index (DAI). (b) Weight loss (%) in each group during treatment.
